# Combining a Being Imitated Strategy With IBT Improves Basic Joint Attention Behaviors in Young Children With ASD

**DOI:** 10.3389/fpsyt.2021.784991

**Published:** 2022-01-07

**Authors:** Birgitta Spjut Janson, Mikael Heimann, Felix-Sebastian Koch

**Affiliations:** ^1^Department of Psychology, University of Gothenburg, Gothenburg, Sweden; ^2^Infant and Child Lab, Division of Psychology, Department of Behavioural Sciences and Learning, Linköping University, Linköping, Sweden

**Keywords:** autism spectrum disorder, early intervention, being imitated, joint attention, intensive behavior treatment

## Abstract

In the present study, we examined how an initial being imitated (BIm) strategy affected the development of initiating joint attention (IJA) among a group of children newly diagnosed with autism spectrum disorder (ASD). One group received 3 months of BIm followed by 12 months of intensive behavior treatment (IBT) which equaled treatment as usual whereas a second group received IBT for the entire 15-month study period. We utilized two measures of IJA: an eye gaze and a gesture score (point and show). IJA did not change during the first 3 months of treatment, nor were any significant between-group differences noted. However, at the end of the 15-month-long intervention period, the BIm group used eye gaze significantly more often to initiate joint attention. No significant change was noted for the gesture score. These results suggest that an early implementation of a being imitated strategy might be useful as less resource intensive but beneficial “start-up” intervention when combined with IBT treatment as a follow-up.

## Introduction

Autism is a neurodevelopmental disorder characterized by impairments in communication and social interaction, along with a restricted repertoire of activities and interests (1). One example of an early developing communication and social interaction skill found to be problematic for autistic children is joint attention, an ability that signifies that a child has developed a capacity to coordinate attention between a social partner and a proximal object or is able to use eye gaze or gestures to establish a moment of triadic attention between him/herself, another person (e.g., a parent), and an object or event (2). A child's ability to follow gaze and to respond to bids for joint attention from others are important both for language and early social-cognitive development [e.g., (3, 4)]. In typical development the first steps to master joint attention are usually observed toward the end of the first year and joint attention is often described as an important developmental milestone [e.g., (3, 5–7)]. Thus, it is of much relevance that several studies have shown that a delayed or altered developmental trajectory of joint attention is one of the earliest problems reported for children with autism [e.g., (8, 9)].

Although joint attention is comprised by both the ability to respond to joint attention bids (RJA) and the ability to initiate joint attention bids (IJA) we focus here only on the latter since IJA has been found to be especially delayed or protracted in children with autism [e.g., (10, 11)]. Joint attention is commonly first observed by a child's eye gaze responses or through gestures such as pointing or showing, abilities that are known to promote learning and communication in incidental situations (12). Difficulties to develop the ability to initiate joint attention will thus have a negative effect on autistic children's daily learning opportunities which makes it critical to target IJA in interventions for young autistic children (3, 4).

In a relatively recent meta-analysis, Murza et al. (13), present support for joint attention interventions in young children with autism spectrum disorder (ASD). A joint attention intervention implies a training of any aspect of sharing attention with a partner about an object, event, or mutual interest. Two different intervention approaches were identified, a general developmental approach that include social interactive strategies [e.g., (14, 15)] and a more focused developmental approach [e.g., (16, 17)]. Murza et al. (13) reported that all 15 reviewed randomized experimental studies demonstrated a statistically significant treatment effect size despite differences in treatment administration, e.g., dosage and design. However, this meta-analysis also revealed that there is limited evidence supporting long-term effects of interventions aiming to develop joint attention.

Imitation has been highlighted as a promising way to promote social behaviors that build up joint attention skills. Of special interest are reports showing that children with autism increase their social motivation as a consequence of being imitated (18–22). Imitation recognition increases children's awareness of being the object of other's social attention, a first step on the road to develop joint attention skills [see (23)]. As an example, Escalona et al. (18) reported that children with autism specifically increased their tendency to initiate social behaviors after only a brief being- imitated intervention. A recent review by Contaldo et al. (24) concludes that a “being-imitated strategy” seems to be generally successful in increasing the social competence and play skills of autistic children. Not only in experimental paradigms but also as part of clinical treatment programs. Thus, this strategy has become more and more accepted since Nadel's first studies and is today included in many intervention programs aimed at children with autism and their parents [e.g., (16, 17, 25–27)].

In the study, we use a being imitated (BIm) strategy in conjunction with a comprehensive program that represented the preferred method (treatment as usual, TAU) at the participating Habilitation clinical center for children with ASD. A comprehensive program is a manualized and broader treatment program that aims to target all or almost all areas deemed important for children with ASD (28). In the literature those programs are often categorized as either Applied Behavior Analysis (ABA) (29) or Intensive Behavior Treatment (IBT) (30). Meta-analyses have revealed that IBT programs generally are effective in promoting medium-effect-size gains in intellectual function, language development, acquisition of daily living skills, and social functioning (31, 32). Of special interest here is that imitation on demand for a long time has been included in programs built on behavioral theory as an ability that needs to be trained in order to improve a child's learning skills [e.g., (33)] but imitation *per se* is not usually the focus since the programs have a much broader scope. IBT is the dominating intervention strategy in Sweden (34, 35) and it usually entails that parents and preschool teachers jointly carry out the training of targeted areas (e.g., imitation, communication, or verbal skills).

The present study examined the effects of two treatment programs. The first one is a novel program that combines an initial 3-month long BIm intervention with 1-year of IBT treatment (Novel = BIm+IBT). The second program used only IBT during the whole 15 months period (TAU = IBT only). Thus, all children received treatment over the same treatment period, they were also allocated randomly to one of the two treatment programs, and were all newly diagnosed with autism according to DSM-IV-TR (36).

The main hypothesis of the study was that the novel program (BIm+IBT) would promote a faster development of behaviors important for initiating joint attention over time than TAU (IBT). Measure of joint attention behaviors were the children's looking pattern (eye gaze) and gestures (pointing and/or showing).

## Method

### Participants

**Figure 1:**
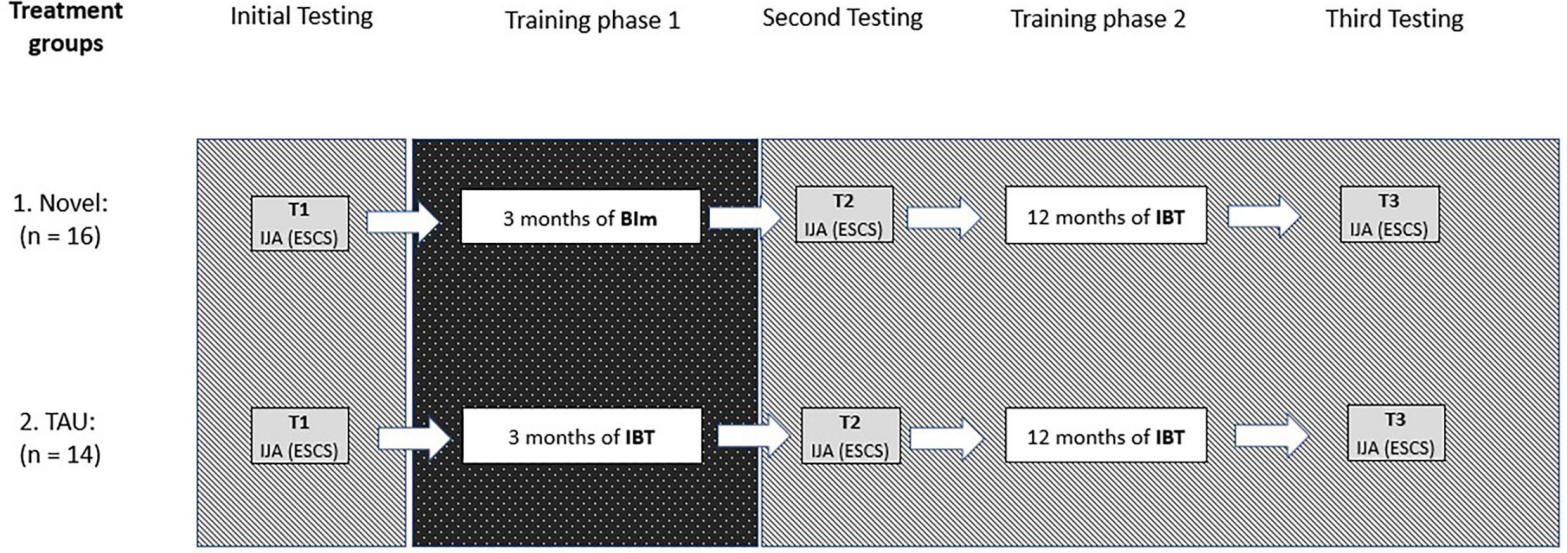
Study design for the participating children (*n* = 30) in the current study. Differences in procedure between the two treatment groups, Novel and TAU, are only found within the dark dotted column. The rest of the procedure was identical (light gray lined area). Attrition in Novel group: *n* = 3, Attrition in TAU group: *n* = 5. Testers for ESCS did not know the group allocation of the child they tested. BIm, Being Imitated; TAU, Intensive Behavior Treatment (IBT); IJA, Initiating Joint Attention; ESCS, Early Social Communication Scale.

All children referred to the Child and Adolescent Habilitation Services in Gothenburg, Sweden, between March 2011 and December 2012, who had a chronological age (CA) of between 24–48 months, and who were recently diagnosed with ASD according to DSM-IV-TR (36) were offered to participate in a randomized control study testing treatment. However, children with severe epilepsy judged to hinder therapy were excluded. After initial testing at T1, the experimenter picked an envelope (prepared by the administrator for blinded randomization) that contained the group assignment, either a novel treatment program with BIm for 3 months followed by IBT for 12 months, or a comparison group that received treatment as usual (TAU = IBT) for the whole 15-month intervention period (see Figure 1).

The current study is a follow-up of a previous study that examined the development of language and social domains from T1 to T2 (Figure 1) (37). The current sample consists of thirty children (see Table 1) that (1) fulfilled the basic inclusion criteria, (2) followed through with training phases 1 (either BIm or IBT) and 2 (IBT for 12 months), and (3) completed our assessment of joint attention, the Early Social Communication Scale (ESCS; 37) both before the treatment commenced (T1) and after the first training phase (T2). Here, we add data for a long-term follow-up (T3) with a specific focus on initiating joint attention. Of the 30 children that qualified for the follow-up study 25 were boys, with an average chronological age of 40.9 months (*SD* = 6.2, range: 26 – 49 months). A majority of the children (*n* = 26) lived with both parents, while four children lived in single-parent households. Parental education was as follows, primary education (i.e., 9 years in school) *n* = 11 mothers and 14 fathers, secondary education (12 years in school) *n* = 8 mothers and 4 fathers, tertiary education (bachelor or master's degree) *n* = 11 mothers and 12 fathers.

**Table 1:** Baseline characteristics of the participating children at start, comparing data for T1 for all children in the sample (*n* = 30) and comparing data for T1 excluding data for children who did not participate at T3 (attrition: *n* = 8) between the two treatment programs: Novel (BIm + IBT) and TAU (IBT only).

	**Sample Group^b^**				**Included in final analysis Group^b^**			
	**Novel**		**TAU**		**Novel**		**TAU**	
	***n*** **=** **16**		***n*** **=** **14**		***n*** **=** **13**		***n*** **=** **9**	
	**M**	**SD**	**M**	**SD**	**M**	**SD**	**M**	**SD**
Age (months)	42.6	6.1	39.0	5.9	41.8	6.5	36.9	6.1
Mental age^a^	20.6	6.7	20.8	9.0	20.6	6.7	19.0	10.0
Expressive language (PEP-R)	5.1	5.0	10.6	10.3	4.2	3.1	8.2	8.9
Expressive language (VABS-II)	14.8	9.2	16.6	9.2	12.9	7.4	14.1	8.5
Receptive language (PEP-R)	7.8	8.4	10.9	10.8	6.4	6.8	8.1	9.9
Receptive language (VABS-II)	15.4	14.6	19.21	12.1	11.4	6.0	14.9	7.3
Gender (F/M)	2/14		3/11		2/11		1/8	
Two-parent families	12		14		10		9	

a *Estimated with Bayley;*

b *All comparisons between groups are non-significant*.

All participants diagnosis were based on a thorough neuropsychiatric work-up using the following clinically validated instruments: The Diagnostic Interview for Social and Communication Disorders (38), Autism Diagnostic Review-Revised (39), Autism Diagnostic Observation Schedule (40), and the Social Communication Questionnaire (41). All clinical staff had extensive time as professionals with both typical and autistic children and were certified in each assessment procedure.

### Treatment Allocation

As already stated, the children were randomized to either a novel program that combined BIm and TAU or to TAU for the whole intervention period (Figure 1). The novel program meant that the children received an imitation-based intervention (BIm) for the first 3 months (from T1 to T2) followed by IBT for the remaining 12 months (from T2 to T3). Our second program, TAU, entailed IBT for the whole 15-month period (from T1 to T3). Both groups had a brief pause of 2–4 weeks after T2 and before continuing with IBT for one year. On average, T2 was conducted 4.9 months (SD = 1.1 months) after T1, and T3 was conducted 12.5 months (SD = 1.3 months) after T2. The actual length of the intervention and of the follow-up period did not differ between the two treatment programs (all *p* > 0.4).

### Background Measures

At T1, before randomization and before treatment commenced the children's language levels were evaluated through two subscales from the Psychoeducational Profile, third edition [PEP-R; (42)] and from an interview with the preschool teachers using two subscales from the Vineland Adaptive Behavior Scales, second edition [VABS-II; (43)]. For the sake of clarity these instruments were also used at T2 representing data that has been published elsewhere (37). Mental age was estimated with the Swedish version of the Bayley Scales of Infant and Toddler Development, third edition (44).

### Measure of Initiating Joint Attention (IJA)

To measure initiating of joint attention (IJA) at T1, T2, and T3, we used the Early Social Communication Scale (ESCS, 37), a structured assessment in which the experimenter presents toys. The toys used in the procedure were strictly selected due to manual descriptions and were exclusively used in the test situation. The experimenter and the child were both seated opposite to each other on either side of a table in a room specially prepared to for the test procedure. The assessment took ~12–14 mins and was videotaped. For our present study, we used only the ESCS tasks that measure IJA (45): eye contact and alternating eye gaze constitutes our eye gaze measure while point and show constitutes our measure of gestures relevant for initiating joint attention bids. Eye contact was noted if the child held an inactive toy and looked at the tester while alternating gaze was coded whenever the child looked back and forth between the tester and an active object.

During the ESCS, the experimenter presented one toy at a time. All children were assessed three times, before the intervention commenced (T1), after three months (T2) and finally 1 year later when the intervention ended (T3). The ESCS was coded from video tapes recorded during the test sessions independently by one research assistant and two master's students in psychology. All three coders were first trained using reference material from typically developing children and proceeded to coding the current material once they were proficient with the infant material (inter-rater reliability ≥ 0.90). The intra-class coefficients between the three coders for the present study material indicated a strong agreement (range, 0.88–0.97). The coders were blind to the aim of the current study, to the children's study treatment group, and to the test phase (T1, T2 or T3) in which the recording was made.

### Novel Treatment (Being Imitated)

The Being Imitated (BIm) intervention was new and unknown to the participating preschool teacher who carried out the intervention. BIm is based on theories and therapeutic strategies mainly formulated by Nadel et al. (20, 46) and Nadel (47) but for the present study, a Swedish manual was developed [Spjut Jansson, (48)]. Adhering to the manual meant that the trainer (a) followed the child's attentional cues, (b) allowed the child to choose the course of the interaction and use of materials, and (c) provided intensified opportunities for the children to engage in activities that are like those in which typically developing peers engage. The imitation procedure was implemented by trained preschool teachers (see below for details on preschool teachers' training), who held training sessions with the children for 30 mins each day over 12 weeks. During these sessions, the adult imitated all behaviors exhibited by the child (except those judged to be harmful or to cause self-injury to the child). The purpose of this procedure is to establish reciprocity by providing the child with an opportunity to show his or her own communication skills and to learn about the social world (49).

All sessions were conducted in a room of the child's preschools, with only the child and the preschool teacher present. Following the procedure of Nadel et al. (50), two sets of identical toys were used to provide opportunities for synchronic imitation. Toys were selected with consideration of each individual child's developmental level and fine motor repertoire, following selection principles to standardize the object variation. One-third of the toys were new to the child (novelty was expected to increase the child's interest), one-third were familiar (e.g., flashlight and a doll), and one-third were selected with the aim to promote object manipulation (e.g., balls, cars, and blocks). The use of familiar toys aimed to reduce any initial resistance and/or anxiety in the child (51), while the offer of commonly used toys was intended to accelerate interest in and skills at using similar toys as other children. For each child a room at the preschool was chosen that was secluded and used in a restricted and limited manner. The selected room enabled the teacher to exclusively attend to the child without any delays or disturbances or interferences from other children or colleagues. Prior to the study, seven experienced clinicians were trained in the method by two of the authors (BSJ and MH) and one experienced colleague in order to be able to support and supervise the preschool teachers. The preschool teachers were similarly trained by the supervisors from the Habilitation Services. They were also filmed for 10–15 mins at the start of the intervention and were thereafter regularly evaluated by two experts (BS and an experienced colleague). Daily protocols and diaries were also used to check treatment fidelity.

All sessions were conducted by the child's preschool teacher, except for one session each week that was conducted in cooperation with a trained supervisor. This supervised session was intended to check treatment fidelity through online supervision and thus increase the preschool teacher's compliance with the BIm manual. The supervised sessions also provided an opportunity to discuss issues that occurred during the treatment sessions with the children.

### Treatment as Usual

Intensive Behavior Treatment (IBT) constitutes the treatment of choice at the participating Habilitation Services and is seen as TAU in the context of this study. This is a manualized comprehensive program, with a curriculum mainly built on insights from ABA (52, 53). IBT uses discrete trial training plus strategies, such as reinforcement, prompt, and prompt fading, with the aim of errorless learning. The manual instructed the preschool teacher and parents to move over time from discrete trials to naturalistic training as the children develop the desired skills. The implemented training was evaluated after each session. Thirteen skills were targeted with specific exercises, with at least six skills covered each day. The training was expected to take 20–25 h each week, with the parents responsible for 10 h and the preschool teachers for 15 h. The parents and preschool teachers participated in supervised sessions lasting 1–1.5 h twice a month. At the beginning, workshops were provided with the aim of teaching the parents and preschool teachers the necessary theoretical knowledge and strategies.

Both parents and preschool teachers completed written reports describing their daily use of exercises and time spent training in order to assess treatment fidelity to the planned program. Parents and preschool teachers also underwent supervised training and treatment fidelity checks by clinicians or special educators who were experienced with the method, on average twice a month.

### Planned and Executed Training

For the Novel group, the plan called for each child to spend 2.5 h per week training with the “being-imitated strategy” during the first 3 months. The mean training time reported by the preschool teachers was 2.2 h per week (SD = 1.0 h) during the first 12 weeks. Over the following 12 months, these children received IBT, that is treatment as usual. They received 20–25 h a week of training provided by parents or preschool teachers, which was in accordance with the planned amount of training for each child. For the TAU group, during the first 12 weeks, the plan called for the children to undergo 15 h per week of training, and the children actually received an average of 14.4 h a week (SD = 2.5 h). During the last 12 months of IBT, both parents and preschool teachers reported 20–25 h of weekly training, also in accordance with the planned time.

### Statistical Analysis

First, treatment effects in the eye gaze score were examined with a mixed-design analysis of variance (ANOVA) with repeated measures across time-points with three levels (T1 to T3) and between group measure interventions (Novel vs. TAU). Then effects on gestures are examined with the same model that was used with eye gaze. We report ${\text{h}}_{\text{p}}^{2}$ for the ES of the factors included in the model. These models were run using IBM SPSS, version 26.0.0.0. To enable comparisons with other studies, the ES for between-group differences were calculated using Hedge's *g* (54). Following the method of Hedges and Olkin [see also (55)], Hedge's g was corrected for small sample size, reducing the effect size by about 4%. Durlak (55) further suggests taking account for pre-test effect sizes when calculating effect size for post-treatment effects. Hence, the effect sizes for post-tests (at T2 and T3) were adjusted for the difference at the previous testing time-point (e.g., adjusted ES_T2_ = ES_T2_ – ES_T1_).

### Ethics

This study was conducted according to guidelines laid down in the Declaration of Helsinki, with written informed consent obtained from a parent or guardian for each child before any assessment or data collection. All procedures involving human subjects in this study were approved by the Regional Ethical Review Board of Gothenburg (418-10, 2010).

## Results

The initial study sample of the current study was *n* = 30 children with the aim of analyzing longitudinal changes in initiating joint attention behavior over the time points. However, there is attrition of *n* = 8 children (3 in the Novel Treatment group and 5 in the TAU group, see Attrition section below for details). Thus, the following analyzes are based on *n* = 22 children, with *n* = 13 in the Novel treatment group and *n* = 9 in the TAU group.

Eye gaze (eye contact and alternating eye contact) and gestures (pointing and showing) were the dependent variables of interest. Eye gaze was more common than gestures (Table 2) at all three time points. Changes in behavior were analyzed with a 3 (time-points T1, T2, and T3, as the within-participant factor) by 2 (Novel vs. TAU as the between-participant factor) repeated measures ANOVA.

**Table 2:** Mean frequencies for the basic building blocks—eye gaze and gestures—measuring Initiation of Joint Attention (IJA) at start (T1), after 3 months (T2) and when the intervention ended (T3) after 15 months.

	**Novel**		**TAU**	
	***n*** **=** **13**		***n*** **=** **9**	
	**M**	**SD**	**M**	**SD**
**Eye gaze (Eye contact and alternating eye contact)**				
T1	11.23	15.25	10.56	15.18
T2	8.77	9.40	15.22	15.42
T3	25.15	13.16	16.11	14.13
**Gestures (Pointing and showing)**				
T1	2.38	3.64	1.33	2.60
T2	2.15	4.71	0.44	0.73
T3	2.08	3.20	0.56	1.33

*Two Treatment Groups: (1) Novel = Being imitated (BIm) combined with Intensive Behavior Treatment (IBT), and (2) TAU = Intensive Behavior Treatment (IBT)*.

### Changes in Eye Gaze Score

**Figure 2:**
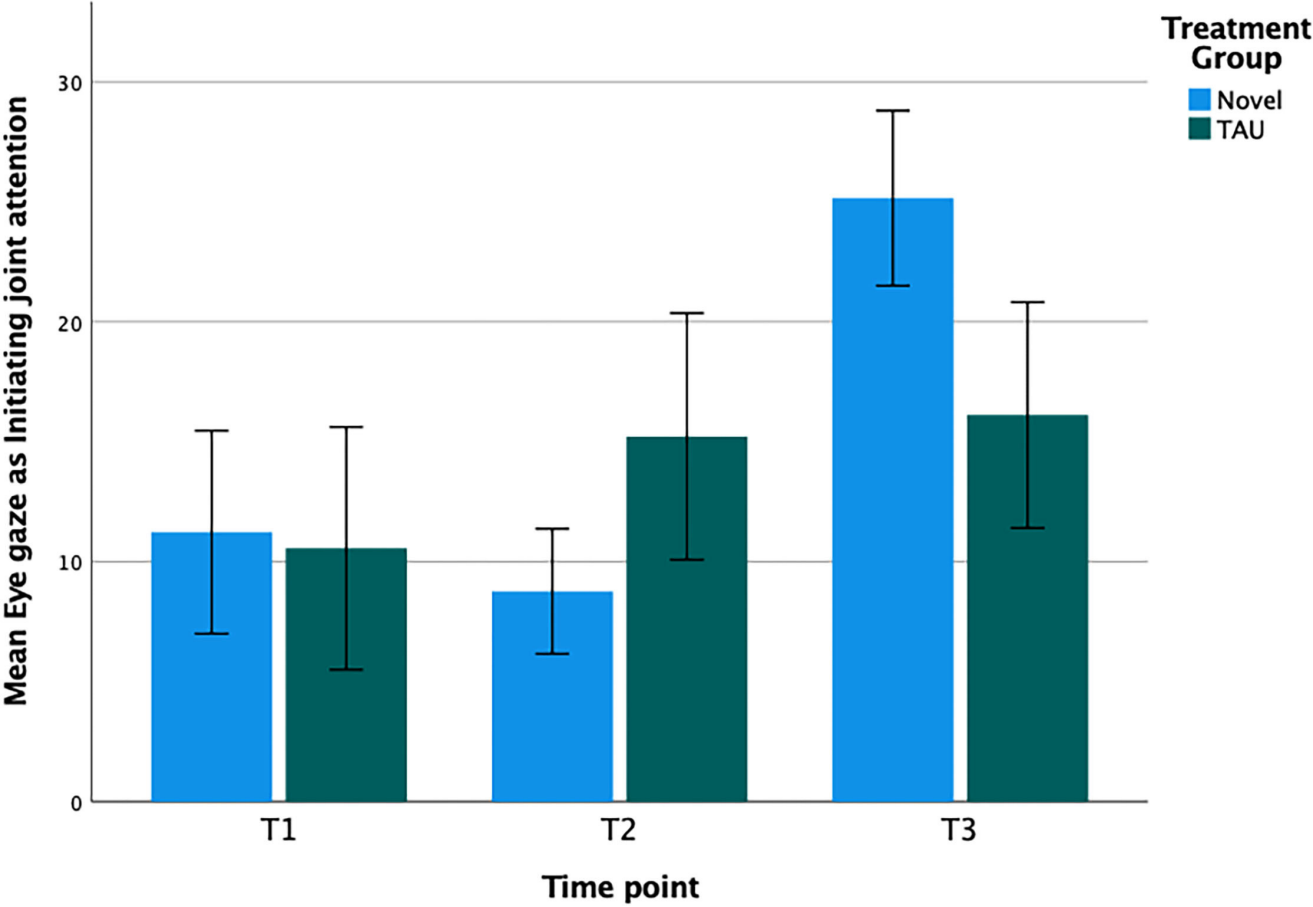
Mean number of observed eye gaze scores with error bars displaying standard error of the mean from T1 to T3 for the Novel group and the TAU group, respectively.

For the eye gaze score (Figure 2), we detected a significant effect of time [*F*_2, 40_ = 6.78; *p* = 0.003; ${\text{h}}_{\text{p}}^{2}$ = 0.25] as well as an interaction between time and treatment [*F*_2, 40_ = 3.58; *p* = 0.037; ${\text{h}}_{\text{p}}^{2}$ = 0.15], but no between-group differences [*F*_1, 20_ = 0.05; *p* = 0.83; ${\text{h}}_{\text{p}}^{2}$ = 0.002]. Tests of within-participant contrast indicated that the eye gaze did not significantly differ between T1 and T2 [*F*_1, 20_ = 0.16; *p* = 0.69; ${\text{h}}_{\text{p}}^{2}$ = 0.008] but significantly increased from T2 to T3 [*F*_1, 20_ = 12.03; *p* = 0.002; ${\text{h}}_{\text{p}}^{2}$ = 0.38]. The interaction between time and treatment was non-significant for T1 to T2 [*F*_1, 20_ = 1.71; *p* = 0.21; ${\text{h}}_{\text{p}}^{2}$ = 0.08] but was significant from T2 to T3 [*F*_1, 20_ = 5.07; *p* = 0.036; ${\text{h}}_{\text{p}}^{2}$ = 0.20]. The eye gaze increased from T2 to T3, an effect that was carried by the observed increase in the novel group. The adjusted effect sizes (Hedge's g) were-−0.53 for T2 and 1.10 for T3, however, only the second effect size was significant.

### Changes in Gesture Score

**Figure 3:**
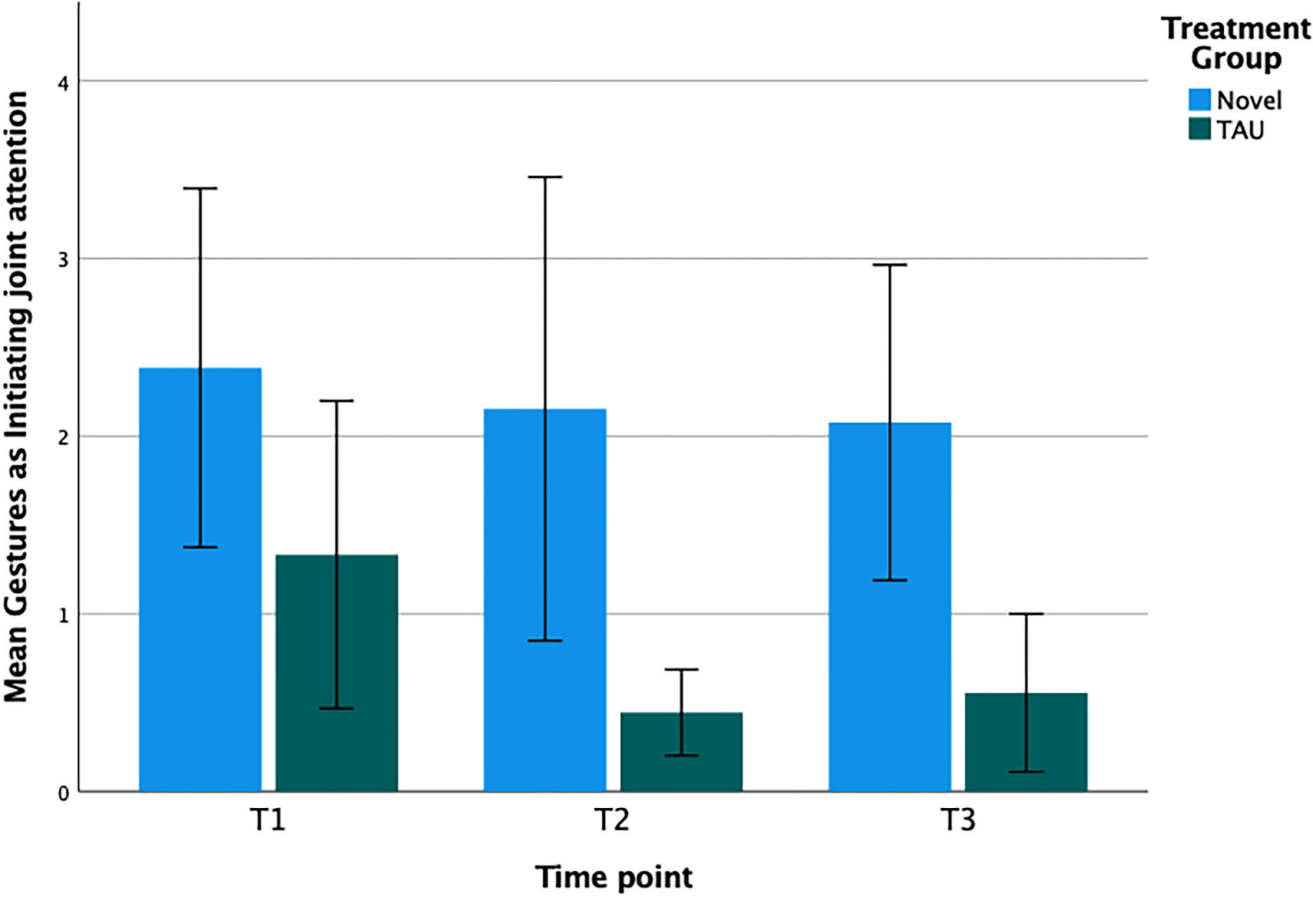
Mean number of observed gestures scores with error bars displaying standard error of the mean from T1 to T3 for the Novel group and the TAU group, respectively.

For the gestures score, we detected no significant effect (Figure 3) in the analyzes over time-points, the interaction between time-point and treatment, or for the observed between-group difference (F values <2.25; *p* > 0.16). Adjusted effect sizes (Hedges *g*) were 0.13 for T2 and 0.11 for T3, but none were significant.

### Attrition

As pointed out above, data for eight children could not be included in the final analyses. Due to technical issues for 2 children (one in each group) during the testing of ESCS at T2 no material was available for analysis. Further, 6 children (2 in the Novel group and 4 in the TAU group) declined to participate in the testing of ESCS at T3.

As the attrition rate in our study was 26 %, we examined possible effects due to attrition closer. No differences in eye gaze and gesture behaviors were found when comparing T1 and T2 scores between children that could be included in the final analysis (*n* = 22) and children that could not (*n* = 8), all *p*'s > 0.60. However, the chronological age of the attrition group (*M* = 43.9 months, *SD* = 3.2) was significantly higher than the chronological age of the included children (*M* = 39.8 months, *SD* = 6.7), *t*_(25.34)_ = 2.22, *p* = 0.035, equal variances not assumed. Overall, the two final intervention groups were not statistical different from each other at T1 on chronological age (see Table 1), language measures (see Table 1), or IJA measures (independent t-test for eye gaze: *p* = 0.92, gestures: *p* = 0.46, see also repeated-measure analyses above).

## Discussion

Here we describe a randomized controlled intervention study performed in a group of young children who were newly diagnosed with autism. The results showed that children who received a Novel package that included a focused being imitated (BIm) intervention (2.2 h weekly during training phase 1) followed by Intensive Behavior Treatment (IBT) equaling treatment as usual (TAU) (20–25 h weekly during training phase 2) showed increased ability to use eye gaze to initiate joint attention compared to the children who received TAU only (15–25 h weekly) over the complete 15-month study period. Initiating joint attention (IJA) was measured on three occasions: before initiation of training, shortly after the end of the first training phase and finally after the second training phase (i.e., after 15 months of treatment). Group comparisons revealed no significant between-group differences before the intervention started or after the first training phase. However, testing after the second training phase demonstrated a significant improvement in eye gaze measures (eye contact and alternating gaze) but not for gestures (point and show) for the Novel intervention (BIm+IBT) compared to TAU (IBT only). This finding suggests that an intervention that builds on a being imitated strategy promotes the development of some of the behaviors needed for young children with autism to initiate joint attention bids.

It is interesting that the intervention found effects over a 1-year period (between T2 and T3) but not any short-term effects from (T1 to T2). This was a bit unexpected since positive short-term effects have been reported in the literature [e.g., (56)]. On the other hand, similar findings to ours have also been reported. For instance, Kaale et al. (57) found a similar pattern following an intervention conducted by non-specialist trainers (preschool teachers). It is worth noting that studies relying on specialist-mediated interventions seems to be more effective in promoting joint attention skills as measured with ESCS [e.g., (16, 58)].

The significant effect emerged when comparing the intervention groups between 3 months (T2) and 15 months (T3), which is the period during which both groups received IBT, the TAU program. Compared to TAU only, the Novel intervention showed higher gains in one of our joint attention measures, with a large effect size. Specifically, the eye gaze score significantly increased for the children in the Novel group, meaning that they displayed an increase of behaviors, such as simultaneously holding an inactive toy and looking at the tester or alternating their gaze between the tester and an active object. On the other hand, gesture, our second measure of initiating joint attention—a summary score of pointing and showing—did not increase for either group. The Novel intervention group did improve in some aspects of IJA (i.e., eye gaze) but not what Mundy considered high level IJA (12), pointing and showing. It is hard to know why but one possibility is that high level behaviors take longer time to develop. They might also need more extensive experience of social interactions.

Both groups received the same treatment during the second training phase, but nevertheless showed different increases in eye gaze after this time-period. It is possible that initiating joint attention skills requires a type of scaffolding other than what is usually provided by ordinary IBT strategies. Contaldo et al. (24) suggest that the being-imitated strategy might be more salient to children with ASD, since the offered contingencies (objects and task) are more predictable and familiar, and thus require less anticipatory skills. Moreover, Nadel (21) concluded that for children with autism, responding to the experience of being-imitated indicates an altered level of recognition of time and structures.

With regards to the eye gaze score, the Novel group showed a large effect size. In a recent meta-analysis, moderate effect sizes were reported from studies that aimed to increase joint attention skills among children with autism (13). They reported an overall Hedges *g* effect of 0.35, which is lower than the presently determined effect size noted for the Novel group at T3 in our study (ES = 1.10). Our present finding of increased initiating joint attention with a large effect size is in line with the suggestion by Mundy et al. (59) that many children with ASD have the capacity to develop joint attention. More recently, Contaldo et al. (24) have highlighted the possibility that focused being-imitated training could influence the neural networks involved in social cognition. Thus, the difference between BIm and IBT (which also uses imitation exercises) may be based on the neural social reward system that is activated by a being imitated interaction [e.g., (60–62); but see (63) for a different view]. In IBT (64, 65), behaviors are concretely rewarded to increase children's willingness to repeat their behavior. On the other hand, in the being imitated intervention, the social reward arises from the sharing of the same emotional state or bodily movement (24).

The main finding of the present long-term evaluation confirmed our hypothesis that BIm, provided with a mean weekly intervention duration of only 2.2 h, promotes the development of eye gaze behaviors that constitute early initial building blocks for IJA. It must be emphasized that this effect was based on a combination of two interventions, a combined treatment program in which an initial 3-month BIm phase was followed by 12 months of IBT/TAU with a training intensity of 20–25 h per week. One possible explanation for the improved effect of the combined treatments might be that the initial implementation of a focused intervention such as BIm increase “the precursors of joint attention skill” in children with ASD. It has been suggested that BIm complements or scaffolds the benefits that a child can gain from a comprehensive IBT program (24, 37). The results from the present study indicate that combing two different programs, one focused (BIm) and one comprehensive (IBT) might be beneficial for developing generalized joint attention skills among young children with ASD.

### Strengths and Limitations

Eight children in our study could not continue their treatment after T2. This attrition seriously affected the power of the study. This is especially relevant to the TAU group, in which one third the children did not participate or had incomplete data sets at T3, compared to one-fifth of the children in the Novel group.

The present study needs to be replicated in a larger group of children to explore moderators of the treatment effect, and in order to better understand possible predictors of children's development of joint attention skills. As the group of children diagnosed with ASD is rather heterogenous a considerable variation between children might be expected, even in joint intention skills such as eye gaze. Despite the small sample size and expected variation a significant result for the being imitated intervention was found. Future studies should validate the current finding and might be able to identify the optimal intervention length, e.g., the number of weeks and the number of sessions per week.

Strengths of this study include the long-term design that includes evaluation of a group of young children recently diagnosed with ASD. The validity of the results is increased by our use of an established operationalized assessment procedure. Another strength is the control of leakage—none of the children had previously received a being imitated intervention or any other behaviorally based treatment prior to the study.

## Conclusions

The present results show significant improvements of eye gaze behaviors that constitute early initial building blocks for IJA in a group of children who received 3 months of being imitated treatment followed by a year of IBT, compared to a group of children who received 15 months of TAU alone. These findings indicate that a combination of interventions, including a being imitated strategy, can facilitate joint attention development among children with ASD. BIm was performed by preschool teachers with a 30-min daily training session (averaging 2.2 h a week), while in the first training phase the TAU training averaged 14.4 h a week and included training both at the children's preschools and at home with their parents. Even though the being imitated group received training of a lower intensity during the first training phase, this group showed greater improvement at the end of the 15-month study period.

## Data Availability Statement

The raw data supporting the conclusions of this article will be made available by the authors, without undue reservation.

## Ethics Statement

The studies involving human participants were reviewed and approved by Regional Ethical Review Board of Gothenburg, Sweden (418-10, 2010). Written informed consent to participate in this study was provided by the participants' legal guardian/next of kin.

## Author Contributions

The study was conceived by BS and MH. Data was collected by BS and analyzed by F-SK. The manuscript was written by BS, MH, and F-SK. All authors approved the final version.

## Funding

BS was supported by grants from Health and Habilitation, Region West Sweden; Queen Silvia's Jubilee Fund, Stockholm; Jerringfonden, Stockholm; and the Petter Silverskiöld Fund, Gothenburg. The work of MH was supported by research grants from the Swedish Council for Working Life and Social Research, Stockholm, Sweden (Grant 2008-0875) and the European Science Foundation Cooperation in Science and Technology Action (ESF COST Action) BM 1004 Enhancing the Scientific Study of Early Autism (ESSEA).

## Conflict of Interest

The authors declare that the research was conducted in the absence of any commercial or financial relationships that could be construed as a potential conflict of interest.

## Publisher's Note

All claims expressed in this article are solely those of the authors and do not necessarily represent those of their affiliated organizations, or those of the publisher, the editors and the reviewers. Any product that may be evaluated in this article, or claim that may be made by its manufacturer, is not guaranteed or endorsed by the publisher.
